# Self-Healing Sulfonated Poly(ether ether ketone)-Based Polymer Electrolyte Membrane for Direct Methanol Fuel Cells: Effect of Solvent Content

**DOI:** 10.3390/polym15244641

**Published:** 2023-12-08

**Authors:** Mae Hwa Tai, Hui San Thiam, Shiau Foon Tee, Yun Seng Lim, Lip Huat Saw, Soon Onn Lai

**Affiliations:** 1 Lee Kong Chian Faculty of Engineering & Science, Universiti Tunku Abdul Rahman, Sungai Long Campus, Jalan Sungai Long, Bandar Sungai Long, Kajang 43000, Selangor, Malaysia; mhtai96@gmail.com (M.H.T.); teesf@utar.edu.my (S.F.T.); yslim@utar.edu.my (Y.S.L.); sawlh@utar.edu.my (L.H.S.); laiso@utar.edu.my (S.O.L.); 2 Centre for Advanced and Sustainable Materials Research, Universiti Tunku Abdul Rahman, Sungai Long Campus, Jalan Sungai Long, Bandar Sungai Long, Kajang 43000, Selangor, Malaysia

**Keywords:** proton exchange membrane, SPEEK, PVA, solvent, self-healing

## Abstract

Proton exchange membranes (PEMs) with superior characteristics are needed to advance fuel cell technology. Nafion, the most used PEM in direct methanol fuel cells (DMFCs), has excellent proton conductivity but suffers from high methanol permeability and long-term performance degradation. Thus, this study aimed to create a healable PEM with improved durability and methanol barrier properties by combining sulfonated poly(ether ether ketone) (SPEEK) and poly-vinyl alcohol (PVA). The effect of changing the *N*,*N*-dimethylacetamide (DMAc) solvent concentration during membrane casting was investigated. Lower DMAc concentrations improved water absorption and, thus, membrane proton conductivity, but methanol permeability increased correspondingly. For the best trade-off between these two characteristics, the blend membrane with a 10 wt% DMAc solvent (SP10) exhibited the highest selectivity. SP10 also showed a remarkable self-healing capacity by regaining 88% of its pre-damage methanol-blocking efficiency. The ability to self-heal decreased with the increasing solvent concentration because of the increased crosslinking density and structure compactness, which reduced chain mobility. Optimizing the solvent concentration during membrane preparation is therefore an important factor in improving membrane performance in DMFCs. With its exceptional methanol barrier and self-healing characteristics, the pioneering SPEEK/PVA blend membrane may contribute to efficient and durable fuel cell systems.

## 1. Introduction

Direct methanol fuel cells (DMFCs) have emerged as a promising alternative to traditional power generation technologies due to their high energy densities, low emissions, and simplicity [1,2]. The polymer electrolyte membrane (PEM) is a crucial part of a DMFC and must be durable under the harsh operating conditions of fuel cells [3,4]. Nafion membranes, which are composed of a hydrophobic fluorocarbon backbone and hydrophilic sulfonic acid pendant groups, have been widely used as a PEM because of their high proton conductivity, good mechanical strength, and excellent chemical stability [5,6]. The formation of a protonated form of Nafion ${(SO}_{3}^{-}H_{3}O^{+})$ in the presence of water facilitates the membrane to transport protons efficiently [7]. Nevertheless, the hydrophilicity of Nafion simultaneously leads to the undesirable crossover of methanol fuel from anode to cathode. Methanol crossover can result in fuel loss and a significant drop in cell voltage, known as a mixed potential [8,9]. Due to the unresolved problem of methanol crossover, increasing the methanol concentration in anode feed from 2 M to 16 M while using Nafion as the PEM resulted in a 66% reduction in power density. As a result, DMFCs typically use a low methanol concentration, with an optimal 1–2 M [10]. Another problem with PEMs used in fuel cells is degradation. The membrane of a DMFC may suffer mechanical damage such as microcracks and pinholes as a result of repetitive swelling and shrinking during operation [11,12]. These damages allow more methanol to permeate through the membrane, negatively impacting the performance and service life of DMFCs. 

To address the issue of methanol crossover, researchers have investigated various methods of modifying Nafion, such as blending with a low-methanol permeable polymer, incorporating inorganic particles, and coating with a layer that is impermeable to methanol [13,14]. Increasing interest has also been shown in the development of non-Nafion-based membranes to counteract the shortcomings of expensive Nafion [15,16]. Among all the developed alternatives, sulfonated poly(ether ether ketone) (SPEEK) is the most promising due to its adjustable proton conductivity and superior mechanical stability [17,18,19,20,21]. By increasing the degree of sulfonation (DS) of SPEEK, it is feasible to increase its proton conductivity [22]. However, methanol permeability also substantially increases with DS. At sufficiently high DS, the crystallized population of SPEEK is fully disrupted, rendering SPEEK amorphous and susceptible to highly polar molecules of methanol and water [23]. SPEEK with a DS above 70% was found to dissolve completely in methanol solution, while SPEEK with a DS of 100% was dissolved entirely in hot water. Therefore, a DS of SPEEK between 30% and 60% was recommended for DMFC applications in order to strike an appropriate balance between proton conductivity and methanol permeability [18]. 

Given the limited DS of SPEEK, the proton conductivity of a SPEEK membrane is still inferior to that of Nafion. Due to this, SPEEK membrane modification has become an important area of study in fuel cell technology. According to Khan et al., blending SPEEK with sulfonated poly(phenylene) oxide (SPPO) resulted in a significant increase in the proton conductivity of the SPEEK membrane owing to its increased hydrophilicity [19]. Another modification strategy involved blending SPEEK with epoxidized natural rubber (ENR-50) with a DS of 80% at different mass ratios. The highest proton conductivity attained by the blend membranes was 47% higher than pure SPEEK, while the lowest permeability value was 21 times less than that of the Nafion 117 membrane. As a result, the membrane selectivity was found to have increased fivefold [24].

Incorporating inorganic fillers has also been considered as a promising approach to improve the conductivity of SPEEK [25]. SPEEK modified with 10 wt% of silica and 5 wt% of silicotungstic acid (SiWA) successfully enhanced the membrane selectivity by up to 6.5 times compared to pure SPEEK due to its increased conductivity and decreased methanol permeability [26]. Another SPEEK composite membrane incorporated with polystyrene sulfonic acid functionalized micelle templated MCM-41 demonstrated 26% higher proton conductivity and 60% lower methanol permeability than pristine SPEEK, resulting in four times higher electrochemical selectivity [27]. In addition, as described by Chikumba et al., a SPEEK membrane doped with 2.5 wt% titanium silicon oxide (TiSiO_4_) could exhibit comparable ionic conductivity to Nafion 117 [20]. The composite membrane was shown to have 2.7 times higher selectivity and 23.3% higher power output at 80 °C than Nafion 117.

To create a SPEEK membrane that was more impermeable to methanol, T. Yang blended a SPEEK polymer with polyvinyl alcohol (PVA), and the resulting blend membrane with 50 wt% PVA increased in methanol resistance by 70% as compared to pure SPEEK [1]. In addition to its excellent methanol-blocking property, PVA possesses an intriguing self-healing ability that could contribute to addressing the PEM degradation issue. As a smart material, self-healing PVA has been the subject of extensive research in numerous applications, including cell culture, smart interface materials, drug release, tissue scaffolding, and shape memory devices [28].

Recently, the concept of self-healing has been applied to fuel cell applications [29,30]. Nafion, PVA, and 4-carboxybenzaldehyde (CBA) were used to produce a self-healing composite membrane by Li. et al. for DMFC application [31]. The exceptional self-healing capability of the membrane was traced to its highly mobile polymer chains and the reversible hydrogen-bonding interactions between Nafion and CBA-modified PVA. The mechanical characteristics and proton conductivity were also enhanced in the composite membrane In another study, Ng, W. W. et al. constructed a self-healable PEM made of Nafion and PVA using a simple freezing–thawing method to incorporate physical crosslinking into the membrane matrix [32]. In addition to its excellent self-repair capability, the blend membrane showed a 40.33% reduction in methanol permeability compared to the recast Nafion membrane. However, the proton conductivity of the composite membrane was significantly lower than that of the recast Nafion. They then added phosphotungstic acid (HPW) to increase the membrane’s conductivity, and the resultant mixed-matrix membrane recorded a peak power density that was 10.7 % higher than recast Nafion [33]. Additionally, the membrane recovered up to 93% of its initial methanol barrier functions after being damaged, suggesting the potential of the membrane to lengthen the service life of DMFC.

The research and application of a self-healing membrane for hydrogen-powered PEMFCs were also discovered. A novel approach to creating fluorine-free self-healing PEMs involved the formation of complexes between phytic acid (PA) and sulfonated polyvinyl alcohol (SPVA), which was then modified with positively charged 4-(1*H*-imidazol-1-yl)benzenecarbaldehyde (IBZ) [34]. The reversibility of electrostatic interactions within the composite membranes allowed for the healing of damaged membranes, with the healed membranes regaining 50% strain in 30 s at room temperature. Apart from its increased durability, the improved proton conductivity of the composite membrane resulted in a higher maximum power density compared to the recast Nafion.

In light of the concept of self-healing, SPEEK was blended with PVA in this study to examine its self-healing behavior and transport properties. This is the first research on the self-repair capability of a SPEEK-based membrane. In addition, the hitherto unreported physiochemical interaction between DMAc solvents and SPEEK/PVA blend membrane was also investigated in this study. DMAc was chosen due to its relatively weak hydrogen bonding with sulfonic acid groups in SPEEK compared to other solvents, which helps in maintaining the high proton conductivity of the fabricated membrane. At the same time, the DMAc-casted membrane could produce a similarly dense membrane morphology as the other membranes cast with dimethylformamide (DMF) and dimethylsulfoxide (DMSO) solvents, thereby reducing the methanol permeability [22,35,36].

## 2. Materials and Methodology

### 2.1. Materials

Polyoxy-1,4-pheneylyeneoxy-1,4-pheneylene (PEEK, molecular weight of 20,800), PVA (99%, molecular weight of 124,000), sulfuric acid (H_2_SO_4_, 99.99%), and *N*,*N*-dimethylacetamide (DMAc, 99.99%) were obtained from Sigma-Aldrich, St. Louis, MO, USA and used without further purification.

### 2.2. Synthesis of Sulfonated Poly(ether ether ketone) (SPEEK)

First, 5 g of 25 μm PEEK powder was dried overnight at 100 °C in an oven and then dissolved in 100 mL of concentrated H_2_SO_4_ at 60 °C for 4 h. The sulfonation reaction was then terminated by quenching the polymer in cold deionized water, which precipitated yellowish noodle-like strands. The precipitated SPEEK was soaked in deionized water overnight and then thoroughly washed until the pH of the filtrates reached between 5 and 6. Following this, the SPEEK polymer was dried overnight in a vacuum oven at 65 °C to ensure the complete removal of all moisture [37]. The DS of the resulting SPEEK polymer was determined to be 60–65%.

### 2.3. Preparation of SPEEK and SPEEK/PVA Blend Membranes

The SPEEK/PVA blend membranes were prepared by solution casting and oven drying. First, 1 g of SPEEK polymer was dissolved in 9 g of DMAc solution at 60 °C. The DMAc solution was prepared by diluting the pure DMAc solvent with deionized water to obtain solutions of varying concentrations, as outlined in Table 1. For sample SP00, which had 0 wt% DMAc content, hot water at 80 °C was employed to dissolve the SPEEK instead of a DMAc solution. In parallel, the required amount of PVA was dissolved in deionized water at 80 °C to yield a 10 wt% PVA solution. Subsequently, the SPEEK solution and PVA solution were mixed at a weight ratio of 6:4. The resulting mixture was then cast onto a petri dish and dried in a vacuum oven at 60 °C for 20 h. To serve as a comparison, the pristine SPEEK membrane was also prepared using the same procedure, except for the addition of the PVA solution. The pristine SPEEK was denoted as S100, while the blend membranes of SPEEK/PVA were labeled as SPXX, where XX corresponds to the DMAc solution concentration specified in Table 1.

### 2.4. Characterization

#### 2.4.1. Structural Characterization

The chemical structure of the pristine SPEEK and SPEEK/PVA blend membranes was determined using a Fourier-transform infrared (FTIR) spectrometer (Nicolet iS10, Thermo Scientific, Waltham, MA, USA). Spectra were collected in the range of 400 cm^−1^ to 4000 cm^−1^ at room temperature.

#### 2.4.2. Water Uptake and Methanol Uptake

Water uptake of the membranes was determined by first immersing them in deionized water for 24 h at room temperature and then drying them in an oven at 65 °C until their weight remained constant. The calculation of the water uptake was performed using Equation (1).

(1) $$WaterUptake=\frac{W_{wet}-W_{dry}}{W_{dry}}\times 100\%$$

where W_wet_ is the weight of a wet membrane (g), and W_dry_ is the weight of a dry membrane (g).

For measuring the methanol uptake, the same procedure as for water uptake was followed, except deionized water was replaced with pure methanol (24.72 M).

#### 2.4.3. Methanol Permeability

Methanol permeability of the membranes was determined at room temperature using the diffusion method. The membranes were fixed between two compartments containing 2 M methanol solution and deionized water, respectively. Samples were collected at intervals of 1 h from the deionized water compartment, and their methanol concentration was determined using gas chromatography (PerkinElmer Clarus 500, Buckinghamshire, UK). Methanol permeability was then calculated from the slope of the straight-line graph of methanol concentrations against the permeation time using Equation (2):

(2) $$P_{M}=\frac{SVt}{C_{MO}A}$$

where P_M_ is the methanol permeability (cm^2^ s^−1^), S is the slope of the graph (M s^−1^), V is the volume of the compartment (cm^3^), t is the thickness of the membrane (cm), C_MO_ is the initial methanol concentration in the methanol compartment (M), and A is the effective diffusion area (cm^2^).

#### 2.4.4. Proton Conductivity

Proton conductivity was calculated from the membrane resistance, which was measured using a four-point probe (in plane) conductivity cell connected to a potentiostat (ZIVE SP1). The sample was cut into 1 cm × 3 cm strips and sandwiched within the conductivity cell. The current was swept from 0 mA to 15 mA with a scan rate of 25 mA s^−1^. A graph of voltage versus current was plotted, and the resistance of the membrane was determined from the gradient of the graph. The proton conductivity, σ, of the membrane was calculated using Equation (3) and expressed in Siemens per cm (S cm^−1^):

(3) $$\sigma =\frac{L}{RWt}$$

where *L* is the distance between the electrodes (cm), *W* is the width of the membrane (cm), *t* is the membrane thickness (cm), and *R* is the membrane resistance (Ω). 

#### 2.4.5. Selectivity

Selectivity is a measure of how well a membrane balances its proton conductivity and methanol permeability. Equation (4) was used to evaluate the membrane’s selectivity, which reflects its overall performance:

(4) $$\Phi =\frac{\sigma }{P}$$

where Φ is the membrane selectivity, *σ* is the proton conductivity, and *P* is the methanol permeability. 

#### 2.4.6. Self-Healing Property

The self-healing ability of the membranes was tested by puncturing three circular holes in them with a 760 μm-diameter needle and then immersing them in a 2 M methanol solution at 45 °C for 2 days. To compare the self-healing ability quantitatively, a methanol permeability test was performed on the damaged and healed membranes according to the above procedure. In addition, a scanning electron microscopy (SEM) instrument (Hitachi S-3400N, Tokyo, Japan) was used to observe the surface morphologies of the damaged and healed membranes. To make the sample electronically conductive, a thin layer of gold was vacuum-sputtered onto it for 4 min before the SEM analysis. The tensile strength of the optimum blend membrane and the pristine SPEEK was then evaluated both before and after the self-healing using a universal tensile machine (Shimadzu AGS-X, Kyoto, Japan) operating at a cross-head speed of 2 mm min^−1^.

All of the procedures for characterization were carried out three times on each sample, and the average values are presented below.

## 3. Results and Discussion

### 3.1. FTIR

**Figure 1:**
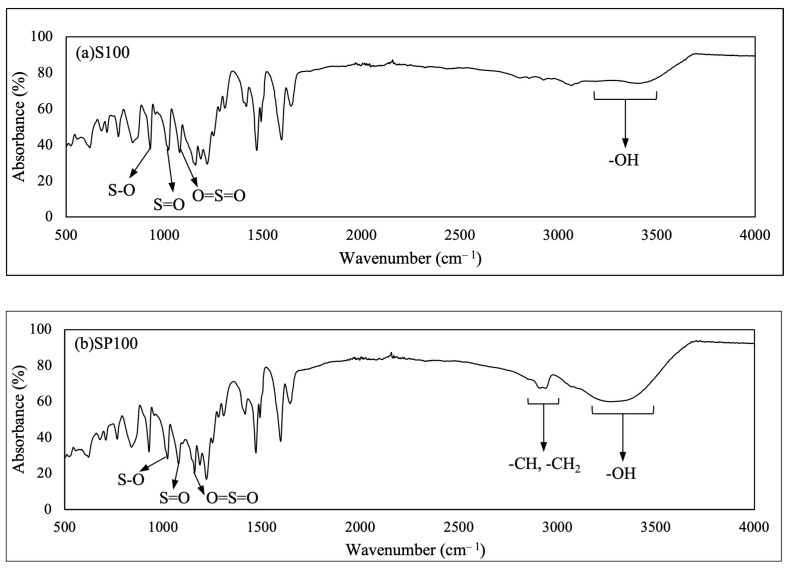
Full FTIR spectra of (**a**) S100 and (**b**) SP100.

Figure 1 shows the FTIR spectra of the pristine SPEEK membrane (S100) and the blend membrane SP100. Absorption bands at 1020 (S=O stretch), 1080 (symmetric O=S=O stretch), 1220 (asymmetric O=S=O stretch), and 841 (S-O stretch) cm^−1^ [38] proved the existence of sulfonic acid groups in the membranes. On the other hand, the broad band between 3000 and 3500 cm^−1^ reflected the O-H vibration [39,40,41]. The characteristic peak of OH stretching in the blend membrane was more intense than in the pristine SPEEK due to the abundance of hydroxyl groups in PVA. In addition, the blend membrane exhibited a more pronounced absorbance band between 2800 and 2900 cm^−1^ because of the increased -CH and -CH_2_ stretching extending from the PVA [42]. These spectroscopic observations validate the inclusion of PVA in the SPEEK network. 

**Figure 2:**
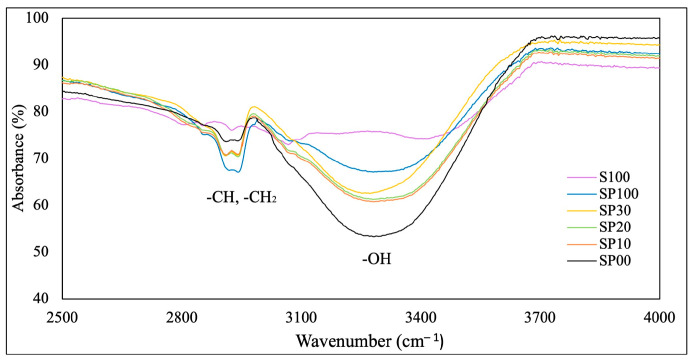
FTIR spectra of S100 and blend membranes in the range of 2500–4000 cm^−1^.

To observe the effect of the DMAc solution, the -OH, -CH, and -CH_2_ peaks in all the membrane spectra were enlarged, as seen in Figure 2. As the concentration of the DMAc solution increased, the intensity of the O-H peak was observed to decrease. This was attributed to the hydroxyl groups in PVA forming hydrogen bonds with the amide groups in DMAc, causing the O-H peak to drop [43]. The strong affinity between PVA and DMAc resulted in a crosslinking effect within the membrane [44]. There was also a noticeable difference in the characteristic peaks of -CH_2_ and -CH between spectra as a result of the different concentrations of the DMAc solution. Reducing the concentration of DMAc during membrane casting decreased the intensity of the -CH and -CH_2_ bands (representing the methyl group in DMAc), showing that the membrane matrix contained less residual solvent after drying [36]. This pattern was easily observed by comparing the band intensities of SP100 and SP00. However, the variations in the -CH and -CH_2_ band intensities between samples SP10, SP20, and SP30 were not prominent due to the minimal disparity in the DMAc concentration. Nonetheless, an overall correlation between the DMAc concentration and the amount of solvent remaining in the membrane was still evident.

### 3.2. Water Uptake and Methanol Uptake

**Figure 3:**
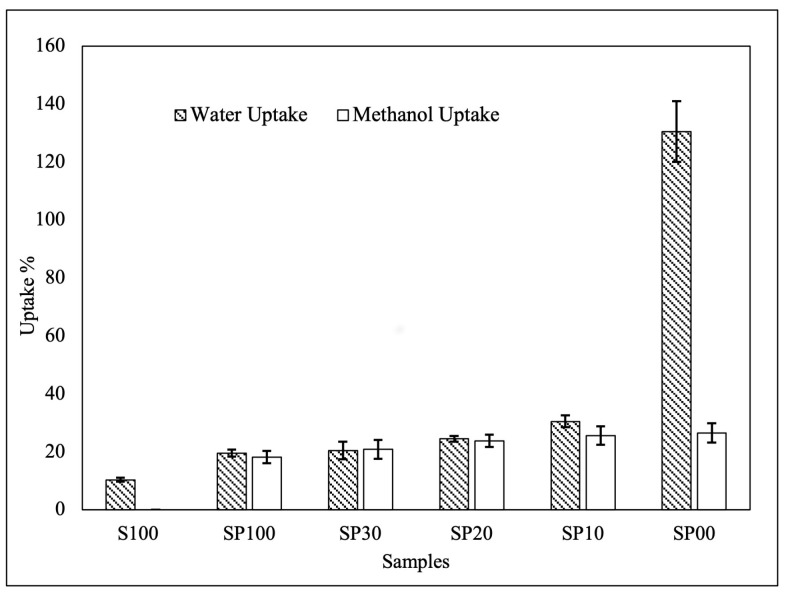
Water and methanol uptake of the S100 and blend membranes.

In an ionomeric membrane, water molecules dissociate acid functionalities, releasing protons, which are then transported by water molecules. Therefore, a PEM must have a sufficient water absorption capacity [24,45]. Figure 3 depicts the water uptake of SPEEK and SPEEK/PVA membranes. Comparing the pristine SPEEK membrane (S100) and the SP100 blend membrane, both of which were prepared with the same concentration of solvent, revealed that SP100 absorbed slightly more water. PVA was present in SP100, and its abundant hydroxyl groups attracted water via hydrogen bonding, resulting in improved wettability of the membrane [8]. 

In addition, the water uptake of the blend membranes was observed to increase with the decreasing solvent concentration, with SP00, which was prepared in the absence of a solvent, exhibiting the most pronounced water uptake. The results corroborated the findings reported by Robertson et al. [46]. Hydrogen bonds were formed between amine groups in DMAc and sulfonic acid groups in SPEEK and between hydroxyl groups in PVA and amide groups in DMAc, resulting in fewer free hydroxyl groups and sulfonic acid groups to absorb water and, thus, a decrease in the hydrophilicity of the membrane. The crosslinking effect caused by hydrogen bond interactions leading to a more compact structure could also explain why SP100 had the lowest water uptake among the blended membranes. This finding suggests that the solvent played a role in increasing the crosslinking density of the membrane.

**Figure 4:**
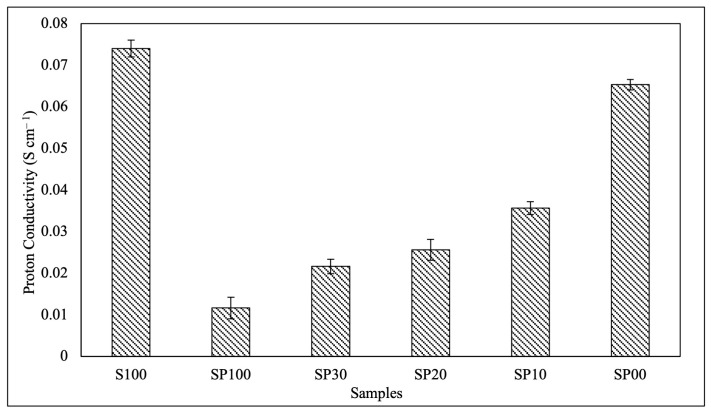
Proton conductivity of the S100 and blend membranes.

For the methanol uptake, the pure SPEEK membrane was dissolved in methanol during the test; therefore, no result was presented. The dissolution of SPEEK in pure methanol was owed to the loss in structural integrity when the DS was more than 60% [10]. In contrast, the mechanical characteristics of the blend membranes were enhanced by the interaction between SPEEK and PVA, which prevented them from dissolving in the methanol solution. The effect of the DMAc solvent concentration on the methanol uptake of the blend membranes is shown in Figure 4. Similar to the trend observed with the water uptake, the methanol uptake decreased marginally as the DMAc concentration increased. The strong hydrogen bonding of DMAc molecules with sulfonic groups in SPEEK and with hydroxyl groups in PVA may have contributed to the reduced evaporation of DMAc during membrane film formation in the oven [35]. Due to the remaining DMAc residue, the membranes became denser and more compact, resulting in a decreased methanol uptake [46].

### 3.3. Proton Conductivity

Figure 4 depicts the proton conductivity of the pure SPEEK membrane and the blend membranes. When comparing the proton conductivity of unmodified SPEEK to that of SP100, the incorporation of PVA into SPEEK significantly decreased the proton conductivity. This result is consistent with the findings of a previous study reported by Sahin, A [47]. The nonconductive PVA lacks proton-conducting groups, and its interaction with SPEEK reduced the amount of accessible sulfonic groups responsible for proton transfer in the membrane, hence decreasing proton conductivity [47,48]. However, the conductivity of the blend membranes improved progressively as the DMAc concentration decreased. This observed trend of proton conductivity corresponded to the results of the water uptake shown in Figure 3. Higher proton conductivity was seen in the blend membranes with greater water absorption, because the water molecules facilitated proton transport. As the DMAc concentration increased, the hydrogen bonding interaction between SPEEK and DMAc reduced the availability of free sulfonic acid groups for proton transport. Simultaneously, the interaction between PVA and DMAc decreased the membrane’s hydrophilicity and, consequently, the number of water molecules as proton carriers, resulting in a decline in proton conductivity [43].

### 3.4. Methanol Permeability

**Figure 5:**
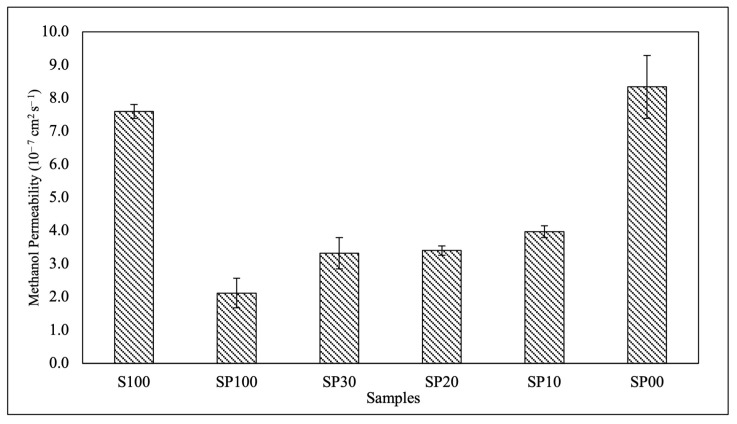
Methanol permeability of the S100 and blend membranes.

Methanol permeability is an important consideration for membrane application in DMFCs, as methanol diffusion may lead to fuel loss and a decline in cell efficiency. The methanol permeability of the blend membranes was evaluated using a 2 M methanol solution, and the results are depicted in Figure 5. Similar trends were observed for methanol permeability and proton conductivity, indicating that methanol molecules and protons share the same transport pathway. As a result, reducing the undesirable methanol crossover will inevitably result in a decrease in proton conductivity. Figure 5 shows that the SP100 membrane, despite having a lower proton conductivity than the pure SPEEK membrane (S100), demonstrated a lower methanol permeability of 74.3%. This proved that the low affinity of PVA for methanol helped to block methanol from passing through the membrane [1]. Furthermore, the interactions between SPEEK and PVA led to the formation of a highly ordered crystalline membrane structure, which increased membrane compactness and impeded methanol transport [48].

**Figure 6:**
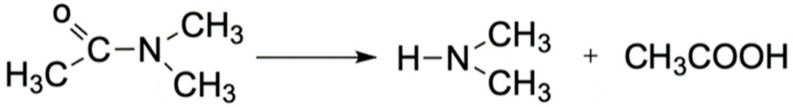
Thermal decomposition of DMAc.

The compactness of the membrane structure was also enhanced by an acid–base interaction between acidic SPEEK and basic dimethylamine (DMA), where the basic DMA formed from the thermal decomposition of DMAc at elevated temperatures during membrane preparations, as illustrated in Figure 6. Acetic acid (CH_3_COOH) was also produced from the free –COCH_3_ groups during the partial decomposition of DMAc [46]. In the presence of acid and water, the decomposition of DMAc could happen even at room temperature. Consequently, as the solvent concentration increased, the greater acid–base interaction in the blend membrane increased its compactness, and this resulted in a decrease in methanol permeability, similar to the trend observed for methanol uptake.

### 3.5. Membrane Selectivity

**Figure 7:**
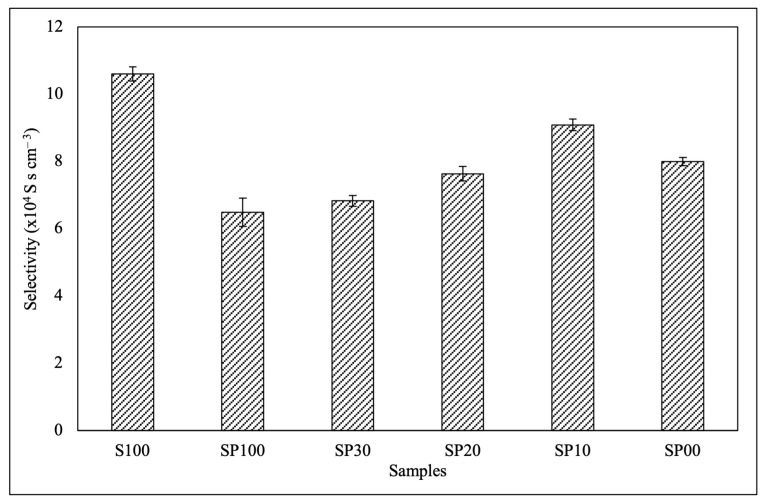
Selectivity of the S100 and blend membranes.

The selectivity of a membrane refers to its ability to allow certain species to pass through while blocking others. When applied to DMFCs, the selectivity of a PEM can be defined as its ability to permit proton transport while preventing methanol transport. Figure 7 shows that, as DMAc concentration was lowered, the selectivity of the blend membranes first increased and then decreased. This was due to the comparative effect of the decrease in proton conductivity and the increase in methanol blocking with the increasing DMAc concentration. Among the blend membranes, SP10 exhibited the highest selectivity. However, its selectivity was still lower than that of pure SPEEK, suggesting the need for further modification in the future to enhance its proton conductivity. The results proved that the DMAc concentration employed during membrane preparation affected the transport efficiency of the membranes.

### 3.6. Self-Healing Property

**Figure 8:**
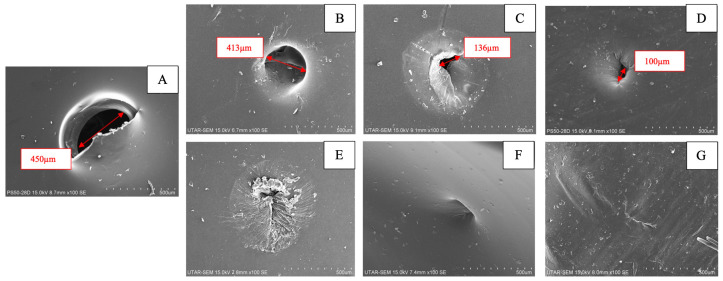
SEM image of (**A**) a damaged membrane, (**B**) SPEEK, (**C**) SP100, (**D**) SP30, (**E**) SP20, (**F**) SP10, and (**G**) SP0 after healing at 45 °C for 2 days.

The self-healing process in this study was conducted at 45 °C, based on the assumption that the operating temperature of a passive direct methanol fuel cell would increase as a result of the heat generated by the chemical reaction [49]. Figure 8 depicts SEM images of the damaged and healed membranes, while Table 2 shows the percentage recovery of the methanol blocking efficiency after healing. All the blend membranes demonstrated better self-healing compared to the pure SPEEK membrane. This could be explained by the fact that the blend membranes contained PVA with abundant hydroxyl groups, which enabled reversible hydrogen bonding within the membrane structure. Furthermore, the presence of water prompted nearby PVA polymer chains to migrate towards the defect site, where they underwent reorganization, forming new bonds and restoring material continuity [37]. Meanwhile, the sulfonic acid groups of SPEEK acted as proton donors and acceptors, allowing water molecules to more easily diffuse into the damaged regions [17]. This enhanced the reorganization of the polymer chains and facilitated the self-healing process.

The self-healing performance was also affected by the solvent concentration. Typically, a higher solvent concentration increases the crosslinking density of a polymeric material, which, in turn, limits chain mobility and results in a more compact network with increased hydrophobicity [50]. Therefore, an increase in the DMAc concentration decreased the ability of the blended membranes to self-heal. This was evident in the observation that SP00 had the best healing function, as indicated by its highest recovery in methanol blocking. Although SP10 showed slightly lower recovery compared to SP00, its superior selectivity compensated for this drawback, making SP10 the most favorable membrane in this study.

An additional metric for evaluating SP10, the optimum membrane, was its tensile strength both before and after self-healing in comparison to the pristine SPEEK (S100). Adding the intrinsically weak tensile strength of PVA to the SPEEK membrane matrix caused the original SP10 (2.43 MPa) to exhibit a lower resistance to tension than S100 (7.34 MPa) [47], as shown in Table 3. Moreover, the increased membrane swelling due to SP10’s higher water uptake indicated a lower crosslinking density and, consequently, reduced mechanical strength. Nevertheless, the SP10 membrane demonstrated a self-healing property that restored 83% of its initial tensile strength. S100, on the other hand, was unable to self-repair and showed a substantial drop in tensile strength, from 7.34 MPa to 3.02 MPa. This further confirmed that PVA contributed to the membrane’s self-healing ability and increased its durability. Notwithstanding this, the blended membrane still needs modifications, such as the incorporation of appropriate inorganic fillers to boost its crosslinking and its overall performance because of its poor tensile strength.

Table 4 summarizes the proton conductivity, methanol permeability, and selectivity of various SPEEK-based membranes as reported in the literature. The selectivity exhibited by the SP10 membrane in the present study is comparable to that observed in other membranes. These findings showed that the SPEEK/PVA membrane is a promising alternative that warrants further research as a PEM in DMFCs.

## 4. Conclusions

In summary, this study provides novel insight into the impact of the solvent concentration on the performance of SPEEK/PVA blended membranes. Strong interactions between the sulfonic acid groups of SPEEK and the amide groups of DMAc reduced the number of free sulfonic acid groups for proton transport, resulting in a drop in the proton conductivity of the blended membranes when the solvent concentration was increased. However, as a result of this interaction, the blended membrane had a more compact structure, making it a superior methanol barrier. In addition, the low affinity of PVA for methanol and the interaction between SPEEK and PVA also contributed to the low methanol permeability of the blended membrane. PVA was also found to be responsible for the self-healing properties of the blended membrane; however, the efficiency of the healing decreased with the increasing solvent content. A higher solvent concentration increased the membrane crosslinking density, which reduced chain mobility and hindered the self-healing process. The SP10 membrane, which was prepared using a 10 wt% DMAc solvent, was shown to be the most effective in this study. The membrane showed the highest selectivity amongst the blend membranes, and more importantly, it could regain 88% of its methanol-blocking capacity and 83% of its tensile strength after being damaged and healed. It is believed that the exceptional self-healing capability of the blended membrane will not only lower membrane costs but also extend the service life of DMFCs.

## Figures and Tables

**Table 1:** Concentration of DMAc solution used during membrane preparation.

Sample	DMAc Solution (wt%)
S100	100
SP100	100
SP30	30
SP20	20
SP10	10
SP00	0

**Table 2:** Recovery of methanol blocking after healing.

Sample	S100	SP100	SP30	SP20	SP10	SP00
Recovery %	7	10	38	61	88	90

**Table 3:** Tensile strength of the original, damaged, and healed S100 and SP10 in wet conditions.

Tensile Strength (MPa)	Original	Damaged	Healed	Recovery (%)
S100	7.34	3.23	3.02	N/A
SP10	2.43	1.01	2.02	83

**Table 4:** Comparison of the performances of various SPEEK-based membranes at room temperature.

Sample	σ (S cm^−1^)	*P_M_* (2 M) (×10^−7^cm^2^ s^−1^)	Φ (×10^4^ S s cm^−3^)	Ref.
SP10	0.036	3.97	5.80	This work
SPEEK-ENR	0.002	0.88	2.40	[24]
SPEEK	0.048	7.45	6.44	[26]
S-DMEA-BPT-PA	0.112	21.70	5.54	[51]
SPEEK-PVDF	0.001	0.65	1.22	[52]
SPEEK-PEEK-alt-BI-3	0.012	1.80	6.60	[53]
SPEEK-PAI	0.040	8.47	4.72	[54]
SPEEK-PAI	0.010	2.50	3.46	[55]
SPEEK-MoS_2_@CNTs	0.042	5.2	3.2	[56]
SPEEK-TiO_2_	0.039	8.44	4.62	[57]

## Data Availability

Data are contained within the article.
